# Current status and perspectives of psychological & behavioral interventional clinical trials (PBCTs) for cancer-related distress: A systematic scoping review of ClinicalTrials.gov

**DOI:** 10.1097/MD.0000000000048196

**Published:** 2026-05-08

**Authors:** Rizwana Amin

**Affiliations:** a Department of Psychology, Effat University, Jeddah, Makkah, Saudi Arabia.

**Keywords:** cancer distress, cancer survivors, caregiver, clinical trials, psychological & behavioral interventions

## Abstract

**Background:**

Cancer is one of the chronic diseases that has a debilitating effect on the mental health of patients as well as their caregivers. Psychological and behavioral interventions are used to alleviate distress symptoms. The present study aimed to review registered clinical trials on psychological and behavioral interventions used to treat cancer-related distress.

**Methods:**

The current study employs the Preferred Reporting Items for Systematic reviews and Meta-Analyses extension for guideline. All interventional clinical trials registered at ClinicalTrials.gov for cancer-related distress were searched as of March 31, 2025. Boolean operators “AND/OR” were used to refine the search. The initial search yielded 341 results, which were reduced to 83 by applying a methodological filter, and only 36 trials fulfilled the inclusion criteria. Only registered trials that use psychological and behavioral interventions to alleviate cancer distress conducted with the adult population were included in the study.

**Results:**

As of September 30, 2025, 36 clinical trials in the ClinicalTrials.gov registry fulfilled inclusion criteria. All registered trials were analyzed qualitatively and quantitatively. Of 36 trials, almost 63.9% have been completed. 75% of studies utilize parallel randomized trials, where 50% of registered trials use Open-label masking. 27.8% of trials utilize the psychoeducational approach to deal cancer-related distress. 61.1% interventions were delivered individually within a clinical setting (66.7%). Moreover, the study identified 6 themes: managing distress, using innovative techniques, personalized care, culturally sensitive approaches, integrated care, and optimizing healing mechanisms.

**Conclusion:**

The progress of inclusive and flexible therapies must continue to advance if psychosocial support is to be completely incorporated into cancer treatment. Patients’ overall well-being can be improved by increasing accessibility and efficacy through interdisciplinary teamwork and culturally sensitive techniques. The findings support improving the psychological well-being of cancer patients and caregivers through policy reforms and research priorities.

## 1. Introduction

Millions of people are diagnosed with cancer each year, making it one of the leading causes of disability and death worldwide.^[1]^ The significant impact of this disease extends beyond physical health, affecting families, communities, and healthcare systems globally.^[2]^ The global burden reached a staggering 19.2 million in 2020, and projections indicate a concerning rise to 21.6 million by 2030.^[3]^ Therefore, cancer-related distress profoundly affects the overall quality of life for patients, leading to emotional, psychological, and physical challenges that can diminish their daily experiences and well-being.^[4]^

Cancer distress encompasses a range of emotional responses, from common feelings of vulnerability and sadness to serious psychological conditions such as anxiety disorders, clinical depression, and traumatic stress.^[5]^ The National Comprehensive Cancer Network^[6]^ stipulates cancer-related distress as “a multifaceted distressing emotional experience” that impairs the efficient strategies for coping with cancer, as well as its treatment and physical symptoms. Literature has identified that almost 30 to 40% of individuals with cancer develop distress at different stages of their disease.^[7,8]^

Cancer-related distress greatly impacts patients’ lives. It leads to treatment avoidance, worsens symptoms, social dysfunction, and reduces survival times.^[7]^ Distress is significantly associated with increased healthcare expenditures and unfavorable survival outcomes in specific patient populations.^[9]^ Therefore, prompt identification of distress is crucial for the timely delivery of effective intervention strategies to manage this issue throughout the continuum of comprehensive cancer care.^[10]^ The biopsychosocial healthcare approach has led to a stronger recognition of behavioral and psychological therapies for cancer distress.^[11,12]^

Specific interventions for cancer patients are developed to handle individual psychological issues, which consist of cancer recurrence fears, together with treatment-related uncertainties, as well as body image changes and end-of-life concerns.^[13,14]^ Various psychological and behavioral interventions exist for alleviating symptoms of cancer-related distress experienced by patients, delivered individually or in groups, or as family interventions or digital programs at any stage of the cancer care.^[15]^ Researchers have designed and conducted a series of rigorous clinical trials to examine the effectiveness of innovative psychological and behavioral interventions^[16–18]^

Psychological and behavioral interventional clinical trials employ diverse approaches, including cognitive-behavioral therapy (CBT) and mindfulness based interventions (MBIs),^[19]^ Supportive-expressive group therapy,^[20]^ Acceptance and commitment therapy (ACT),^[21]^ Psychoeducation and digitally delivered behavioral programs.^[22]^ Clinical trials include a variety of interventions that help cancer patients enhance their emotional coping skills, lessen their distress, and enhance their functioning from the time of their cancer diagnosis to the survivorship phase and the end-of-life stage.^[23]^

The number of Psychological and behavioral interventional clinical trials designed to treat cancer-related distress has increased substantially during recent years because researchers identified better psychosocial needs and improved research approaches. Most of the research has been conducted in high-income countries. The present study aims to evaluate the psychological and behavioral registered clinical trials. The present study examines existing evidence and provides insights to update clinical guidelines, shape research agendas, and advocate for policy changes to enhance the psychological well-being of cancer patients. A more compassionate and equitable model of cancer care can be established by putting evidence-based behavioral and psychological therapies for cancer-related distress into practice.

## 2. Methods

### 2.1. Research design

In this study, the systematic scoping review design was used to examine the characteristics and thematic focus of randomized controlled trials registered on ClinicalTrials.gov. The eligibility criteria, screening, and data extraction plan were guided by the populations–concept–context framework and aligned with Preferred Reporting Items for Systematic reviews and Meta-Analyses extension-Scoping Reviews recommendations^[24]^

### 2.2. Data sources and search

A systematic search was conducted on the ClinicalTrials.gov database for all cancer distress-related clinical trials registered by March 31, 2025. The search was done using the terms “Cancer distress” OR “Burden of Disease” OR Psychological Distress” AND “Psychological Interventions” OR Behavioral intervention” OR “Psychoeducation based therapy” OR “psychosocial therapy.” Duplicate trials were removed. The summary of all results for this search criterion was then read and sorted to verify whether they met the inclusion or exclusion criteria. The registered trials were then read in their entirety before repeating the eligibility verification process.

### 2.3. Data extraction

Registered trials on cancer distress were manually extracted and downloaded from https://clinicaltrials.gov using specified inclusion and exclusion criteria. The dataset for cancer distress clinical trials was limited to include only psychological and behavioral intervention studies involving the adult population conducted up to that date. Moreover, the search included studies across all trial phases to provide a comprehensive overview. Additionally, any registered clinical trials that were terminated, withdrawn, or suspended were excluded from the final analysis. The trials were independently extracted and screened from the published data in the clinical trial registry by 2 reviewers. Essential information was systematically collected to facilitate the analysis, encompassing timeframes, reported results, participant numbers, and primary outcomes.

Initially, 341 clinical trials on cancer distress were identified in the ClinicalTrials.gov database. 258 records were removed from the dataset using automated tools based on the irrelevance of their titles, keywords, trial design, and interventions. From the remaining 83 trails, 20 duplicate records were removed. The remaining 63 studies were screened on primary outcome measures, that is, distress, disease burden, anxiety, depression, and quality of life; 19 studies were excluded at this step. Moreover, the remaining studies were screened based on age group, and all studies conducted with children and adolescents were also excluded. This step reduced the studies to 36. Hence, a total of 36 registered trials were included in this review. A Preferred Reporting Items for Systematic reviews and Meta-Analyses extension flowchart is presented as flow diagram.

### 2.4. Data categories

The following information was collected from each study: current status, participants, interventions, sponsors, estimated enrollment, eligibility criteria (age), study designs, and locations.

According to https://clinicaltrials.gov, the clinical trials were being conducted on different continents. The clinical trials are located in the following regions: Asia, Europe, North America (including the United States, Canada, and Mexico), and other (including Africa, Middle East, Oceania, and South and Central America). Studies with no location reported were excluded from the counts, and studies with multiple locations were counted for each region. Detailed characteristics of the included studies are presented in the Table S1, Supplemental Digital Content

### 2.5. Data analysis strategy

The data were analyzed quantitatively and qualitatively. Quantitative data analyses were performed for registered trial characteristics using Microsoft Excel 365^[25]^ and Statistical Package For Social Sciences v.26.0.^[26]^ Data were expressed as absolute numbers and percentages for categorical variables, and medians and interquartile ranges (IQRs) for continuous variables. Whereas Qualitative data analysis employed a content analysis strategy following Krippendorff^[27]^ approach to identify key themes based on the analytical categories derived from frequencies and percentages of the registered trials.

## 3. Results

### 3.1. Clinical trials characteristics

Among the 36 clinical studies analyzed, the majority were completed (63.9%) and included both male and female participants (66.7%). A maximum of 250 participants were enrolled in the trial, including cancer survivors and caregivers. 75% of registered trials used a parallel study design, predominantly open-label (50.0%), but 83% randomly allocated participants to groups. The primary purpose of these trials was on supportive care (69.4%) for cancer survivors & caregivers (77.8%). Over half of the trials (52.8%) describe the cancer as a general medical condition without specifying the subtype under investigation. Table 1 presents the characteristics of the clinical trials, including clinical trial status, participant numbers, gender, study population, allocation, Study design, masking, disease condition, study purpose, geographical region, and sponsoring institutions.

**Table 1:** Clinical trial characteristics (n = 36).

Clinical Trial Characteristics		f	%
Clinical Trial Status			
	Recruiting	6	16.6
	Not yet recruiting	3	8.3
	Unknown	2	5.6
	Completed	23	63.9
	Enrolling by invitation	2	5.6
Participants			
	Female Only	12	33.3
	Male Only	0	0
	Both (Male & Female)	24	66.7
Enrollment			
	0–250	30	83.3
	251–500	5	13.9
	> 500	1	2.8
Study Population			
	Cancer survivors	28	77.8
	Cancer Caregivers	5	13.9
	Both	3	8.3
Study Design			
	Parallel	27	75.0
	Single Group	3	8.3
	Sequential	6	16.7
Masking			
	Open-label	18	50.0
	Single	10	27.8
	Double	3	8.3
	Triple	3	8.3
	Quadruple	1	2.8
	Not available	1	2.8
Allocation			
	Randomized	30	83.3
	Non-randomized	4	11.1
	N/A	2	5.6
Disease Condition			
	Brain Tumor	2	5.6
	Lung Cancer	5	13.8
	Breast Cancer	4	11.1
	Not specified	19	52.8
	Other	6	16.7
Study Purpose			
	Supportive Care	25	69.4
	Treatment	9	25.0
	Prevention	1	2.8
	Not Available	1	2.8
Region			
	Africa	1	2.9
	Asia	7	19.4
	Europe	12	33.3
	North America	16	44.4
Sponsoring Organization			
	Hospital	5	13.9
	University	21	58.3
	Research institutes	8	22.2
	Other	2	5.6

n = number of individuals.

### 3.2. Intervention used in clinical trials

Many psychological and behavioral interventions have been used in clinical trials included in this review. Among interventions used to address psychological distress among cancer survivors and caregivers are psychoeducational (27.8%) and psychosocial (25.0%) approaches. 61.1% of interventions were delivered individually within a clinical setting (66.7%). Table 2 presents the types, their delivery modes, and formats.

**Table 2:** Interventions and their characteristics used in clinical trials (n = 36).

Clinical Trial Interventions Characteristics		f	%
Psychological & Behavioral Interventions			
	Psychosocial	9	25.0
	Psychoeducational	10	27.8
	Psychoeducational & Interpersonal counseling	2	5.5
	CBT	3	8.3
	*Third Wave CBT (MBCT, ACT)	6	16.7
	+Other	6	16.7
Intervention Delivery Mode			
	Individual	22	61.1
	Group	14	38.9
Intervention Delivery Platform			
	Clinical Setting	24	66.7
	Online	12	33.3
	Internet-based	4	
	Mobile applications	1	
	Telephone	3	
	Videoconferencing	1	
	Remote Care	1	
	Computerized	1	
	Virtual therapy	1	

ACT = acceptance & commitment therapy, CBT = cognitive behavior therapy, MBCT = mindfulness-based cognitive therapy, n = number of individuals.

* Number of trials in third wave of CBT (MBCT=5; ACT= 1).

+ Number of trials in Other (Hippotherapy = 1; mindfulness-based stress reduction = 1; Yoga = 1; Music therapy = 1; stress management = 1; neurocognitive remediation therapy = 1).

### 3.3. Summary of identified key themes

**Figure 1.**
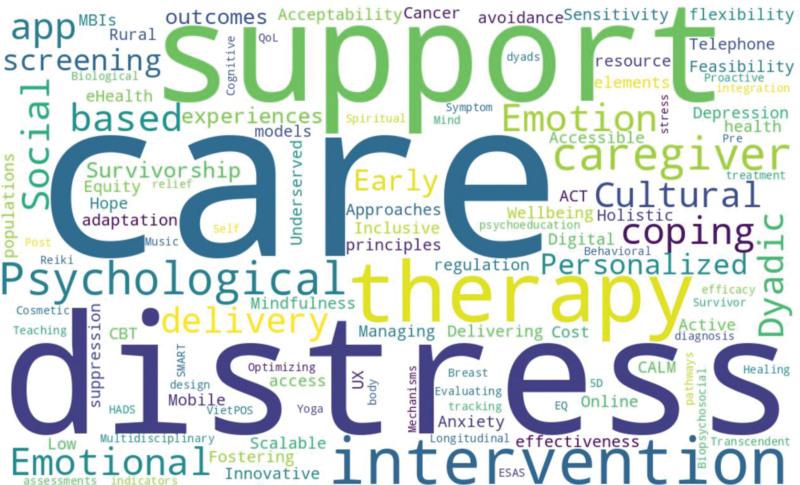
Word Cloud for identified themes.

The present study utilized the Krippendorff^[27]^ approach to analyze registered trials data. The analysis of the evaluated intervention studies identified 6 predominant themes that encapsulate the scope and course of psychosocial and behavioral strategies in cancer care. Table 3 represents identified key themes, associated codes, their types, and descriptions. The word cloud for the identified theme is shown in Figure 1.

**Table 3:** Identified key themes, coding, types of codes, and their description.

Themes	Analytical Category Features	Frequency & Percentages	Description of Analytical Category
Managing Psychological Distress and Fostering Emotional Well-being	Psychological distress, Anxiety, Depression, Emotional coping, Active coping, Emotion regulation, Social support	29 (82%)	A central aim of interventions is to reduce psychological distress, such as anxiety, depression, and social isolation. Moreover, it promotes emotional resilience, hope, and effective coping strategies, e.g., Trial 2, 24
	Hope, Emotion suppression Social avoidance		
Innovative and Scalable Approaches to Delivering Interventions	Mobile app-based therapy, Digital health delivery, Online intervention, App-based MBIs, UX principles, Cost-effectiveness, Feasibility/acceptability, Telephone delivery	20 (57%)	Interventions increasingly utilize technology and evidence-based therapies (CBT, ACT, MBSR) to provide accessible, scalable, and cost-effective mental health care via digital and mobile platforms. e.g., trial 3, 9, 14
	CALM therapy, CBT elements, ACT, Mindfulness therapy, psychological flexibility, eHealth		
Equity, Cultural Sensitivity, and Accessible Inclusive Care	Underserved populations, Cultural adaptation	5 (15%)	By using context-sensitive, low-resource, and community-adapted strategies, the emphasis is on culturally appropriate interventions and closing access gaps for marginalized and diverse people. e,g, trial 13, 22
	Rural access, Low-resource models		
Holistic and Dyadic Cancer Care	Dyadic support, Caregiver psychoeducation, Mind-body integration, Survivor-caregiver dyads	13 (37%)	Interventions address patients’ and their supporters’ psychological issues, integrate caregivers, and encourage mind-body therapies in a dyadic and holistic manner. E.g. trial 6, 34
	Support for caregivers, Pre-diagnosis stress, Breast screening distress, Teaching intervention, Music therapy, Reiki/Yoga, Cosmetic care		
Personalized, Early, and Proactive Survivorship Support	Survivorship care, Self-efficacy, SMART design, Personalized pathways, Early intervention, Multidisciplinary care, Symptom relief, Biopsychosocial outcomes	14 (40%)	Prioritize proactive, personalized care that starts early in the cancer journey, addresses survivorship needs, promotes self-management, and alleviates symptoms through adaptive, multicomponent, biopsychosocial techniques. E.g. trial 8, 22, 2.
	posttreatment distress, Behavioral screening		
Evaluating and Optimizing Healing Mechanisms	QoL assessments (EQ-5D, HADS, VietPOS, ESAS), Longitudinal tracking,	24 (68%)	This research examines both conventional and innovative healing methods, evaluating quality of life, cognitive outcomes, biological indicators, and spiritual or transcendent experiences to determine the efficacy of interventions. e.g. trial 12, 26
	Sleep, Exercise, Cognitive impairment, Fear of recurrence, Psychedelic-assisted therapy, Spiritual/transcendent mechanisms		

One trial appears in multiple themes.

ACT = acceptance & commitment therapy, CALM = cancer and living meaningfully, CBT = cognitive behavior therapy, EQ-5D = EuroQOL, ESAS = Edmonton Symptom Assessment System, HADS = Hospital Anxiety and Depression Scale, MBCT = mindfulness-based cognitive therapy, MBI = mindfulness based interventions, MBSR = mindfulness based stress reduction, n = number of individuals, QoL = quality of life, UX = user experience, VietPOS = Vietnamese Palliative Care Outcome Scale.

First theme, Managing Psychological Distress and Fostering Emotional Well-being, highlights the importance of psychological treatment methods. In all reviewed registered trials, all interventions highlighted the importance of treating psychological distress while boosting emotional wellness as a core objective through dealing with anxiety and depression symptoms together with social isolation and fostering hope and emotional control, and active coping skills. These patterns indicate that patients maintain long-lasting mental challenges while demonstrating the therapeutic power of improving both personal strengths and social networks. A key component of Innovative and Scalable Approaches to Delivering Interventions was the growing use of mobile applications alongside eHealth tools and online CBT/ACT platforms. By leveraging technological advancements, novel approaches to care delivery have been developed that improve accessibility at a reasonable cost and offer adaptable options such as Managing Cancer and Living Meaningfully and mindfulness-based therapy.

Equity, Cultural Sensitivity, and Accessible Inclusive Care highlights the need for healthcare practitioners to develop tailored treatment plans for underprivileged patients from remote locations and across all demographic groups. Furthermore, Holistic and Dyadic Cancer Care indicated a transition towards the inclusion of caregivers and family members, alongside the integration of alternative therapies such as music therapy, yoga, and mind-body integration. Likewise, the next theme, Personalized, Early, and Proactive Survivorship Support, targets adaptive care pathways, such as behavioral screening, survivorship planning, and effective delivery models, that start early in the cancer trajectory.

Last but not least, Evaluating and Optimizing Healing Mechanisms underscores the importance of established and emerging methods, such as the use of biological markers (e.g., cortisol, C-Reactive Protein), standardized quality of life evaluations, and advanced treatments like psychedelic-assisted therapy and spiritual mechanisms to eliminate existential distress. In short, these themes collectively highlight an evolution in the realm of psychological care for individuals affected by cancer. This progression reflects a deeper understanding of the complex emotional and mental health needs of patients, ensuring that their psychological well-being is prioritized alongside their physical treatment.

## 4. Discussion

Current psycho-oncology research needs and knowledge priorities are reflected in the unique primary patterns seen in research on behavioral and psychological trials intended for cancer distress. Prior research has identified high levels of psychological distress with a cancer diagnosis^[28,29]^ Intervention trials continue to prioritize the mitigation of psychological distress, notably symptoms of anxiety, depression, and social withdrawal.^[30]^ Along with alleviation of symptoms, the goal of these therapeutic strategies was to help people develop mental resilience, active coping, and hope.^[31]^ The methods offer a shift from pathology-focused models to positive psychological functioning, aligning with strength-based cancer treatment models.

Nowadays, there is a rising focus on addressing the mental health needs of those who provide care for cancer patients. Growing awareness of caregiver burden has likely drawn more attention to their mental health issues, which include emotional tiredness, worry, and depression as a result of long-term intensive care responsibilities.^[32]^ Along with the cancer survivors, the healthcare emphasis has broadened to encompass caregivers, since cancer impacts a larger psychosocial network. Therefore, psychological and behavioral interventions incorporate relational dimensions, with caregivers involved in therapy. Recent research has identified the deteriorating effects of disease on the family’s support system.^[33]^ Involving caregivers in the treatment model increases treatment efficacy by facilitating the management of caregiving burden.

Enrollment of cancer survivors and caregivers in registered trials includes both sexes and fosters widespread applicability. However, female-only trials indicate gender differences in healthcare-seeking behaviors.^[34]^ This suggests that women are more likely to participate in studies aimed at alleviating distress symptoms and promoting psychological well-being. This is also indicative of the under-recognition of men’s emotional needs, consequently making men resistant to accepting professional help for addressing psychological health issues due to social stigma.^[35]^

In general, registered trials use randomized parallel-group designs to maintain methodological rigor. Since complete masking is hard to accomplish in psychological and behavioral therapies, open-label methods are often used in most clinical trials, which leads to potential performance detection bias^[36]^ especially when outcomes profoundly rely on self-report measures. The trend in health care research is to use multiple assessment methods to minimize bias. To address this, registered trials are focusing on collecting data through biomarkers^[37]^ as well as psychedelic-assisted therapies^[38]^ to meet the biopsychosocial needs of cancer survivors. Most of the registered clinical trials reviewed for the study do not specify a particular cancer type. The researchers used a transdiagnostic approach since different cancer diagnoses show similar signs of psychological distress.^[39]^ This strategy enhances overall research outcomes but overlooks the unique emotional responses elicited by specific cancer types.

Systematic reviews of completed trials of psycho-oncology interventions indicate that both CBT and MBIs have small-to-moderate effects on emotional well-being and quality of life.^[40,41]^ The third wave of CBT emphasizes acceptance, self-compassion, and present-moment awareness, such as mindfulness-based and acceptance-based methods, which are becoming increasingly popular because they align with coping mechanisms for chronic illnesses.^[42]^ Hence, targeted intervention programs will improve treatment efficacy and applicability. Moreover, the interventions tailored for the cancer survivors and caregivers incorporate educational and supportive components.^[43]^ These approaches can be easily integrated into the existing cancer care model.

Moreover, these interventions can be delivered in both individual and group settings. Although clinical settings continue to be the main environment for service delivery, the growing utilization of online platforms signifies a transition towards greater scalability and accessibility,^[44]^ particularly pertinent in the post-pandemic context. Digital interventions provide adaptable and economical solutions, especially for individuals in remote or resource-limited environments^[45,46]^

Most studies were conducted in high-income countries, such as Europe and North America, which limits the generalizability of our findings to low- and middle-income countries, where the cancer burden is substantial. It indicates disparities in research facilities, institutional capabilities, and available research funding.^[47]^ Consequently, this raises concerns about the generalizability of research findings and highlights the lack of appropriate cultural interventions across diverse regions. Prior literature has shown the cultural adaptation of various therapies, such as CBT, MBIs, and ACT, across the globe.^[48]^ According to international mental health studies, the cultural adaptation of the intervention becomes an important factor influencing participant acceptance and intervention success rates.^[49]^

### 4.1. Conclusion

The current review highlights the use of psychosocial and behavioral interventions developed to alleviate cancer-related distress. Patient care interventions integrate structured psychological therapies, digital tools, mindfulness-based strategies, and early supportive care. This approach demonstrates a substantial commitment to enhancing the quality of life for cancer patients and their caregivers throughout the entire care process. The development of sustainable and inclusive strategies that facilitate the accessibility of psychological care for cancer patients is still imperative. To enhance patient engagement and access, psychological support for cancer patients necessitates technological solutions, culturally appropriate design investments, and establishing collaboration across disciplines.

### 4.2. Limitation & future recommendation

The review focused exclusively on registered clinical trials in English from a single registry, clinicaltrials.gov, limiting generalizability. Moreover, it limits the completeness and representativeness of the identified trials registered in the regional or other international platforms. Although Scoping reviews are not routinely registered, this lack of registration reduces transparency and limits reproducibility.

Risk-of-bias assessment using tools like Cochrane risk-of-bias assessment 2.0, or analyses of selective reporting and publication bias, was not feasible because evaluating effectiveness and real-world impact for certain registered trials is limited by ongoing or unpublished results. Additionally, including terminated or withdrawn trials in future analyses could offer a more comprehensive view of the research landscape. The current paper does not include publication status; however, future analyses can incorporate it to provide valuable insights into evidence gaps.

Since this review provides crucial information on test practicality, cultural responsiveness, and patient-focused adaptations, further research should proceed with qualitative studies alongside community-based interventions. Long-term follow-up measures are necessary in research trials to ascertain how long behavioral and psychological effects last. Research on trial design should prioritize diverse inclusion by implementing plans that account for caregiver input, resource constraints, and the population’s cultural demands. The practical implementation of translational research will establish a link between controlled trials and routine clinical practice, thereby ensuring that psychosocial cancer support is accessible to all patients affected by the disease.

## Acknowledgments

The author acknowledges the contribution of independent reviewers (SI & TR) in the data extraction and screening process.

## Author contributions

**Conceptualization:** Rizwana Amin.

**Data curation:** Rizwana Amin.

**Formal analysis:** Rizwana Amin.

**Methodology:** Rizwana Amin.

**Writing** – **original draft:** Rizwana Amin.

**Writing** – **review & editing:** Rizwana Amin.


